# Physical Properties of Blood Are Altered in Young and Lean Women with Polycystic Ovary Syndrome

**DOI:** 10.1371/journal.pone.0167290

**Published:** 2016-11-30

**Authors:** Michael J. Simmonds, Nikki Milne, Kee Ong, Emily Brotherton, Antony P. McNamee, Jarod Horobin, Surendran Sabapathy

**Affiliations:** 1 Menzies Health Institute Queensland, Griffith University, Gold Coast, Queensland, Australia; 2 Bond Institute of Health and Sport, Faculty of Health Sciences and Medicine, Bond University, Gold Coast, Queensland, Australia; 3 Monash IVF, Southport, Queensland, Australia; Université Claude Bernard Lyon 1, FRANCE

## Abstract

Classic features of polycystic ovary syndrome (PCOS) include derangement of metabolic and cardiovascular health, and vascular dysfunction is commonly reported. These comorbidities indicate impaired blood flow; however, other than limited reports of increased plasma viscosity, surprisingly little is known regarding the physical properties of blood in PCOS. We aimed to investigate whether haemorheology was impaired in women with PCOS. We thus measured a comprehensive haemorheological profile, in a case-control design, of lean women with PCOS and age-matched healthy controls. A clinical examination determined similar cardiovascular risk for the two groups. Whole blood and plasma viscosity was measured using a cone-plate viscometer. The magnitude and rate of red blood cell (RBC) aggregation was determined using a light-transmission aggregometer, and the degree of RBC deformability was measured via laser-diffraction ektacytometry. Plasma viscosity was significantly increased in women with PCOS. Blood viscosity was also increased for PCOS at lower-to-moderate shear rates in both native and standardised haematocrit samples. The magnitude of RBC aggregation–a primary determinant of low-shear blood viscosity–was significantly increased in PCOS at native and 0.4 L·L^-1^ haematocrit. No difference was detected between PCOS and CON groups for RBC deformability measurements. A novel measure indicating the effectiveness of oxygen transport by RBC (i.e., the haematocrit-to-viscosity ratio; HVR) was decreased at all shear rates in women with PCOS. In a group of young and lean women with PCOS with an unremarkable cardiovascular risk profile based on clinical data, significant haemorheological impairment was observed. The degree of haemorheological derangement observed in the present study reflects that of overt chronic disease, and provides an avenue for future therapeutic intervention in PCOS.

## Introduction

Polycystic ovary syndrome (PCOS) is the most prevalent endocrine disorder among women of reproductive age [1]. Classic features of PCOS include disordered reproductive, metabolic, and psychological health [2], although the presentation of these factors vary with age. Infertility may be noted during early-to-mid adulthood [3], at which time systematic screening often reveals a metabolic impairment that may have persisted for over a decade. Significant associations have been identified between PCOS and abdominal obesity, hypertension, impaired glucose tolerance, insulin resistance, type 2 diabetes mellitus, and dyslipidaemia [3]. Indeed, the progression from normoglycaemia towards impaired glucose tolerance, and also from impaired glucose tolerance toward type 2 diabetes, is accelerated in PCOS [4]. Given these factors each represent discrete risk factors for cardiovascular disease, it is not surprising that PCOS promotes vascular dysfunction [5], leading to blood flow impairments that precede overt cardiovascular and metabolic diseases [6].

Many of the late-stage complications in metabolic and cardiovascular disorders share a strong positive relation between severity of dysfunction and impairment in blood fluidity [7–9]. While it is well-established that PCOS is associated with impaired vessel health and therefore may explain some of the cardinal features of this disorder, the aetiology of vascular dysfunction in PCOS remains poorly described. Moreover, while blood flow is heavily influenced by the functions of the vascular network, an often overlooked but equally important aspect of tissue perfusion and blood fluidity is the medium *itself*: the physical properties of blood, and especially red blood cells (RBC) are primary determinants of tissue perfusion, nutrient delivery, and blood flow [10]. Indeed, the study of the flow and physical characteristics of blood (haemorheology) has demonstrated that impairments in RBC function may precede the onset, and predict the severity, of various chronic diseases [11]. Many of the factors that impair the physical properties of blood cells–oxidative stress, inflammation, etc–are also strongly related to PCOS [12, 13], thus it is curious that so little is known regarding the haemorheological profile of women with PCOS.

Limited studies have reported that plasma viscosity is increased in young women with PCOS as a function of obesity and the degree of insulin resistance [14], and most likely as a result of increased plasma fibrinogen concentration [15]. It is plausible that whole blood viscosity is also increased in PCOS, given plasma viscosity is an important determinant of whole blood viscosity. Importantly it has been demonstrated that blood hyperviscosity exacerbates vascular dysfunction [16], which may be particularly important in PCOS given its strong association with poor vessel health. In the present study, it was hypothesised that haemorheological parameters would be impaired in women with PCOS, when compared healthy women matched for age and body mass. We observed for the first time that fundamental physical properties of blood are significantly altered in young and lean women with PCOS.

## Materials and Methods

### Participants

Thirteen women with PCOS and thirteen age-matched healthy controls (CON) provided witnessed written informed consent to participate in this study. Individuals with PCOS were diagnosed by their respective fertility or endocrine specialist according to the Rotterdam criteria [17] and subsequently screened by the investigators. Exclusion criteria included: i. medications known to interfere with blood fluidity and haemodynamics (e.g., anti-hypertensives); ii. cigarette smoking within the last 12 mo; and, iii. history of overt cardiovascular, microvascular, respiratory, haematological and/or metabolic disorders (unless consistent with PCOS, e.g., insulin resistance); iv. known endocrine disorders (e.g., Cushing’s syndrome, androgen-secreting tumours, etc.). All participants reported normal thyroid function, while liver and kidney function was determined in consultation with each woman’s general medical practitioner. A list of reported medications for the participants is provided in Table 1. Four women using biguanides reported this use was prophylactic and historic hyperglycaemia was not noted. No participants were using lipid-lowering therapies. Ethinyloestradiols were all combined therapies, including: i. cyproterone acetate 30 mg (n = 1), 35 mg (n = 3), ii. drospirenone 35 mg (n = 2), iii. levonorgestrel 35 mg (n = 2).

**Table 1:** Medication use reported during clinical examination.

	CON (*n* = 13)	PCOS (*n* = 13)
Contraceptive use	8	5
Etonogestrel	4	1
Ethinyloestradiols	4	4
Ethinyloestradiol dose (mg)	30–35	35
Duration of use (yr)	1–6	1–9
Biguanide / other glucose lowering	0 / 0	4 / 0
Other: salbutamol	2	2

Data are absolute frequency values, unless otherwise indicated.

### Experimental design

During the visit to the clinical laboratory, participants underwent a clinical examination. At the completion of the clinical measurements, 10 mL of whole blood was collected into tubes containing 1.8 mg·mL^-1^ of K_2_-EDTA. The experimental protocol was conducted during 2014 in accordance with the Declaration of Helsinki and approved by the Human Research and Ethics Committees of Griffith University and Bond University. Haemorheological assays were conducted within 4-h of collection.

### Clinical Measures

Participants were covered with a light blanket and asked to rest quietly for 10 min. Blood pressure was subsequently measured, before standing height was measured to the nearest 0.5 cm using a stadiometer. Body mass was measured to the nearest 0.1 kg (MC-980MA, Tanita Corporation, Tokyo, Japan).

### Blood preparation

Whole blood was drawn from each sample into a glass capillary tube and packed cell volume (i.e., haematocrit) was determined. The blood was then aliquot, where one portion remained at its “native” haematocrit, while the other portion was adjusted to a standardised (0.4 L·L^-1^) haematocrit using autologous plasma.

### Whole blood and plasma viscosity

Viscosity of plasma, and whole blood at native or 0.4 L·L^-1^ haematocrit, was measured at 37°C and shear rates of 75–1500 s^−1^ using a rotational cone-plate viscometer (0.5 DVII+ with CPE40 spindle, Brookfield Engineering Labs, Middleboro, MA). Haematocrit-to-viscosity ratio (HVR) was calculated for whole blood at native haematocrit by expressing haematocrit (%) to blood viscosity at each shear rate. The HVR has been explored as an index of oxygen delivery effectiveness of RBC based on the rationale that increased numbers of RBC is concurrently beneficial for oxygen transport, but also negatively increases blood viscosity, and therefore resistance to flow [18, 19].

### RBC aggregation measurements

RBC aggregation was determined using a cone-plate shearing system (Myrenne RBC Aggregometer, Myrenne GmbH, Roetgen, Germany). Red blood cell aggregation was measured for two conditions: i. blood samples at native haematocrit; ii. blood samples adjusted to 0.4 L·L^-1^ haematocrit in autologous plasma. Two values that increase with enhanced RBC aggregation were determined: “M_0_”, the degree of RBC aggregation at stasis within 10-s following a cessation of an applied high shear (600 s^-1^); and “M_1_”, the degree of RBC aggregation at a low shear (3 s^-1^) within 10-s following the applied high shear (600 s^-1^). The rate of RBC aggregation at stasis was also measured over a 120 s period, and expressed as the half-time (T_1/2_) of the amplitude measured during this period.

### RBC deformability measurements

RBC deformability was measured using an ektacytometer (Rheoscan-D200, Sewon Meditech. Inc., Seoul, Korea) operating at 37 ± 1°C. Diluted RBC suspensions were transferred into a slit-flow channel (with a height of 200 μm) also stored at 37 ± 1°C. The RBC suspensions were then subjected to varied shear stresses ranging from 0 Pa to ~25 Pa, and a low-power laser produced diffraction patterns that were circular for cells at rest, and became progressively ellipsoidal as RBC deformed (as determined by the elongation index; EI). The subsequent data was used to derive discrete EI across a range of shear stresses (0.5–20.0 Pa). Non-linear curve-fitting enabled the calculation of the following indices: i. the maximum theoretical EI at infinite shear stress (EI_max_), and; ii. the shear stress required for half of EI_max_ (SS_1/2_).

### Statistical analysis

Normality of the data were examined using the Shapiro-Wilk test with visual inspection for kurtosis and skew of the data. Greenhouse-Geisser corrections were applied, when necessary, where an inequality of variance was detected. Differences in the mean values for each group were compared using independent–samples *t*–tests. Blood viscosity and RBC deformability data were compared using a multifactorial ANOVA with repeated measures, to determine whether significant differences in the means existed (Prism 6, Graphpad Software Inc., La Jolla, CA). Data are reported as mean ± standard error unless otherwise stated.

## Results

The physical characteristics of participants are presented in Table 2. No significant differences were detected between groups for mean age, body mass, body mass index (BMI), or blood pressure at rest. Haematocrit was significantly lower in women with PCOS when compared with CON (p = 0.016).

**Table 2:** Basic characteristics women with polycystic ovary syndrome and healthy controls.

	CON (*n* = 13)	PCOS (*n* = 13)
Age (yr)	31 ± 11	29 ± 8
Body mass (kg)	64.5 ± 10.4	68.7 ± 14.8
Body mass index (kg·m^-2^)	23.8 ± 4.2	24.9 ± 5.4
Systolic blood pressure (mm Hg)	117 ± 7	112 ± 13
Diastolic blood pressure (mm Hg)	72 ± 9	73 ± 9
Haematocrit (L·L^-1^)	0.41 ± 0.03	0.39 ± 0.01^*^

Data are mean ± standard deviation. PCOS, polycystic ovary syndrome. CON, healthy controls.

*, significantly different p < 0.05.

### Blood and plasma viscosity

**Fig 1:**
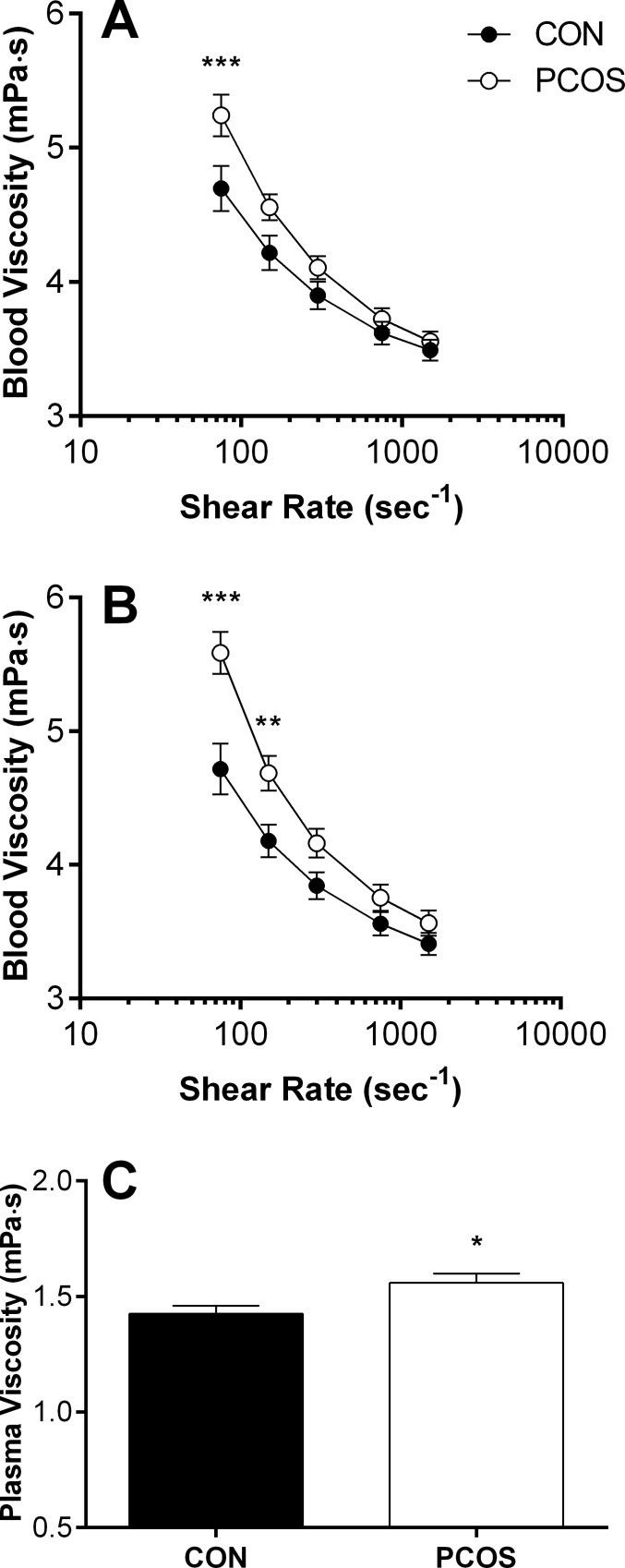
Viscosity of blood at native (A) or standardised haematocrit (B), and plasma (C) for women with polycystic ovary syndrome (PCOS) and healthy women matched for body mass and age (CON). Data are mean ± SEM. ***, PCOS significantly increased, p < 0.001. **, PCOS significantly increased, p < 0.01. *, PCOS significantly increased, p < 0.05.

The typical shear-thinning behaviour–i.e., decreased viscosity with increased shear rate–of blood is observable in Fig 1A and 1B. In whole blood samples at native haematocrit, blood viscosity was increased at lower-to-moderate shears for women with PCOS when compared with CON, reaching significance at 75 s^-1^ (p < 0.001). Blood adjusted to a standardised haematocrit demonstrated a similar trend, although the differences between groups were more pronounced, with blood viscosity significantly increased at 75 (p < 0.001) and 150 s^-1^ (p = 0.004) in women with PCOS. Women with PCOS also presented with ~10% higher plasma viscosity (Fig 1C; p = 0.027).

### RBC aggregation

**Fig 2:**
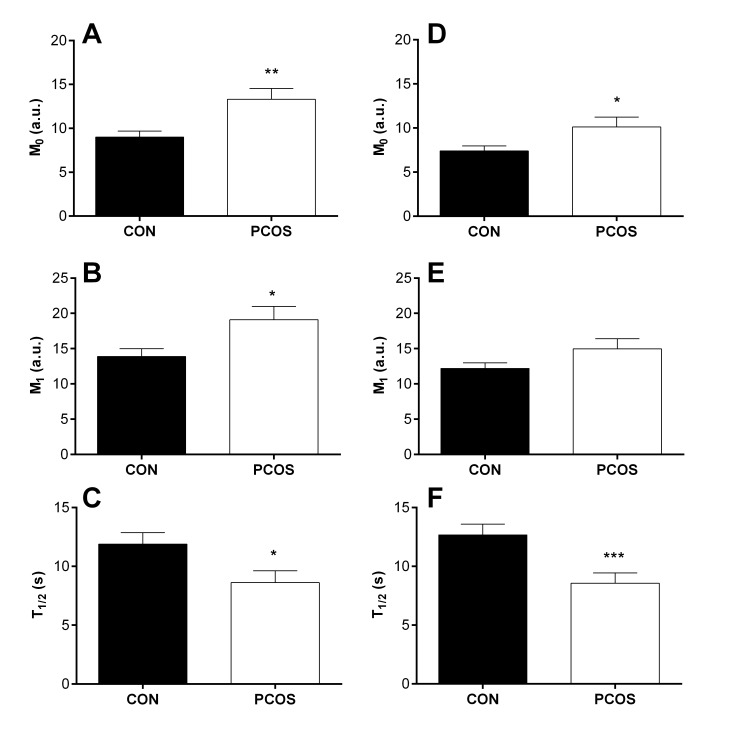
The magnitude and rate of red blood cell (RBC) aggregation for women with polycystic ovary syndrome (PCOS) and healthy controls (CON). RBC aggregation was increased at stasis (M_0_; A) and under low shear (M_1_; B) in native haematocrit samples. When haematocrit was standardised, M_0_ was significantly increased (D), although M_1_ was no longer significantly different (E). The half-time required for maximal RBC aggregation (T_1/2_) was increased when determined in native (C) and standardised haematocrit (F). Data are mean ± SEM. ***, PCOS significantly different, p < 0.001. **, PCOS significantly different, p < 0.01. *, PCOS significantly different, p < 0.05.

The magnitude and rate of RBC aggregation for women with PCOS and CON is presented in Fig 2. When blood was measured at native haematocrit (Fig 2A–2C), the magnitude of RBC aggregation was significantly increased at stasis (i.e., M_0_; p = 0.006) and under low shear conditions (i.e., M_1_; p = 0.025). The rate of RBC aggregation, as determined by the half-time required for maximal RBC aggregation at stasis (i.e., T_1/2_), was significantly decreased (i.e., worse) for women with PCOS (p = 0.029).

To account for the pro-aggregating effects of haematocrit, the magnitude and rate of RBC aggregation was determined for samples using standardised haematocrit (Fig 2D–2F). The M_0_ parameter remained significantly higher (p = 0.042) in women with PCOS, although there was no longer a statistical difference between groups in the M_1_ parameter (p = 0.056). The T_1/2_ also remained significantly decreased for women with PCOS (p = 0.003).

### RBC deformability

**Fig 3:**
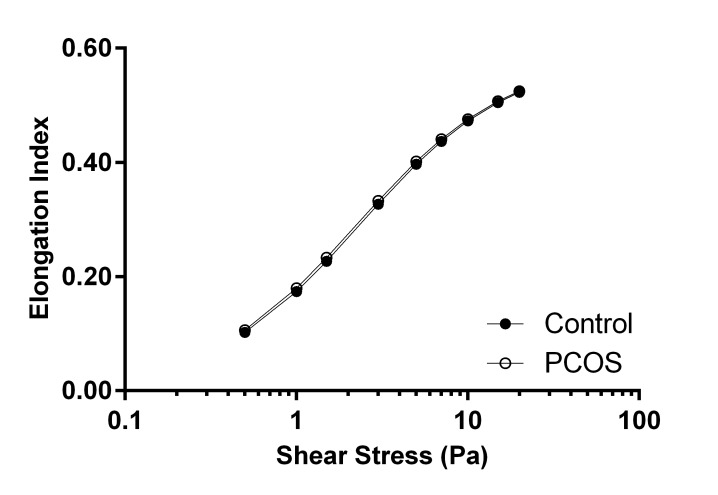
The elongation index (i.e., deformability) of red blood cells (RBC) determined at discrete shear stresses between 0.5 and 20.0 Pa for women with polycystic ovary syndrome (PCOS) and healthy age and body mass matched controls (CON). Data are mean ± SEM.

The relationship between RBC deformability and shear stress was typically sigmoidal, where the elongation index (a measure of RBC deformability) increased with the rise in shear stress (Fig 3). RBC deformability was not different between women with PCOS and CON at any shear stress. The EI_max_ was not significantly different (CON 0.585 ± 0.005 vs. PCOS 0.585± 0.004; p = 0.926), and the SS_1/2_ also did not significantly differ between groups (CON 2.32 ± 0.08 vs. PCOS 2.29 ± 0.10; p = 0.864).

### Haematocrit-to-viscosity ratio

**Fig 4:**
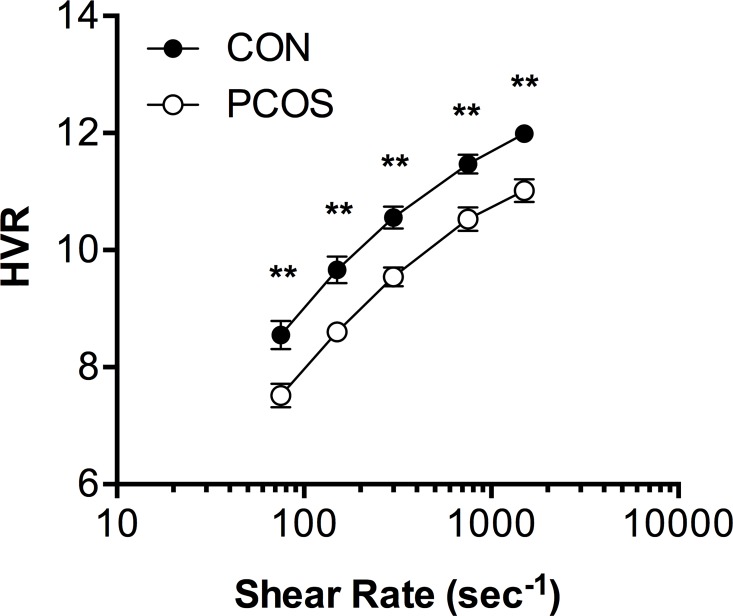
The haematocrit-to-viscosity ratio (HVR) at discrete shear rates (75–1500 s^-1^) for women with polycystic ovary syndrome (PCOS) and healthy age and body mass matched controls (CON). Data are mean ± SEM. **, PCOS significantly increased, p < 0.01.

The RBC oxygen transport effectiveness (i.e., HVR) is illustrated in Fig 4. It was found that HVR for healthy women (CON) were comparable to earlier reports [18, 19], and women with PCOS presented with significantly decreased HVR at all shear rates (main effect p < 0.001). At the highest measured shear rates (750 and 1500 s^-1^), HVR was 8% lower in PCOS; the reduction became greater at 300 (10%), 150 (11%) and 75 s^-1^ (12%). Thus, HVR fell a greater amount with decreasing shear rate in PCOS when compared with CON.

## Discussion

Based on basic clinical data, the women with PCOS in the present study superficially appeared to have similar cardiovascular health profiles as the healthy controls. It was revealing, therefore, that distinct differences were observed in haemorheological health. Blood viscosity at lower-shear rates was significantly elevated in PCOS (Fig 1A), and this could not be explained due to differences in baseline haematocrit; when blood samples were standardised for haematocrit, differences in lower-shear blood viscosity remained significant (Fig 1B). Various metabolic and cardiovascular disease states are associated with impaired blood fluidity, and are clustered as “hyperviscosity syndromes” [20]. Interpreting increased blood viscosity in a given population is complex, given that healthy individuals demonstrate a transient increase in blood viscosity during exercise, and increased blood viscosity during exercise is paradoxically associated with improved cardiorespiratory fitness [21]. Major distinguishing features that separate whether increased blood viscosity is detrimental include the state of vascular health, and the duration of exposure to elevated blood viscosity. Indeed, an acute and reversible increase in blood viscosity is likely to upregulate mechanosensitive pathways in (healthy) endothelial cells, facilitating nitric oxide production, whereas chronically elevated blood viscosity and/or increased shear stress combined with endothelial dysfunction is associated with adverse outcomes [22]. The increased blood viscosity observed in young women with PCOS is likely a sign of early disease processes that contribute to heightened cardiovascular risk [16], given considerable evidence has accumulated over the past decade that these women are highly susceptible to endothelial dysfunction [6], in addition to venous thromboembolism [23], coronary artery calcification [24], and increased carotid intima-media thickness [25]. In the case that the vascular network is structurally damaged or less functional in PCOS, the chronically increased blood viscosity observed in the present study may result in a “vicious cycle” whereby autoregulation of vasomotor tone may not be able to compensate for impaired physical properties of blood, which may explain, in part, the elevated cardiovascular risk associated with PCOS [16]. Interventions aimed to reduce blood viscosity are of potential therapeutic value, but this aspect of cardiovascular health has been overlooked until recently [22].

Plasma viscosity was significantly increased for women with PCOS (Fig 1C) in the present study. Vervita et al., [14] reported that plasma viscosity was not different between a cohort of PCOS women and healthy controls, although obese women with PCOS presented with significantly increased plasma viscosity compared with lean PCOS and healthy controls. In the present study, no significant relation was detected between BMI and plasma viscosity for women with PCOS, although this may be due to the narrow range of BMI values of the present women. Moreover, differences in the ethnicities of the present women and those of the previous study [14] may explain discordant findings, as ethnicity of women with PCOS has been demonstrated to influence the profile of cardiovascular risk factors [26]. Elevated plasma viscosity in PCOS is likely the result of increased plasma fibrinogen concentration, given the viscosity of plasma is determined by its protein content, and fibrinogen’s relatively large molecular weight and fibrous structure make it the most important plasma protein in this regard [27]. A possible explanation for increased plasma fibrinogen in PCOS may originate with increased inflammatory processes [13], decreased fibrinolysis [28], and as an acute phase reactant, fibrinogen concentration is likely increased due to the associated prothrombotic state in PCOS [29]. Two studies have reported positive and significant relations between plasma fibrinogen and plasma viscosity [14] in PCOS, although the weak-to-moderate strength of these correlations indicate that other, currently unknown, factors also contribute to the increased plasma viscosity. It should be noted that the increased plasma viscosity contributes, in part, to the elevated blood viscosity of the present women with PCOS (discussed previously). The significance of elevated plasma viscosity may be highlighted in the context of type 2 diabetes: patients with elevated plasma viscosity (defined as > 1.45 cP) present with significantly increased systolic and diastolic blood pressure when compared with patients with normal plasma viscosity levels [30]; this finding may be of particular importance in the context of the vascular complications associated with PCOS.

Another primary determinant of low-shear blood viscosity is RBC aggregation, and in the present study, it was found that the magnitude and rate of RBC aggregation was significantly increased (i.e., worse) in women with PCOS. Increased RBC aggregation has not been previously reported for women with PCOS, and represents an altered balance between disaggregating (e.g., electronegative membrane surface charge) and proaggregating (e.g., plasma protein concentration) determinants of aggregate formation [10]. While evidence is limited, two studies indicate that serum sialic acid concentration is not altered by PCOS, which may suggest that RBC negative charge is also not influenced by PCOS. A probable explanation for the increased RBC aggregation in PCOS is the proaggregating properties of plasma fibrinogen (increased in PCOS, per previous discussion), which promotes aggregate formation via depletion interactions that ultimately draw RBC into close proximity, creating rouleaux [31]. The increased rate of RBC aggregate formation (i.e., T_1/2_) in the present study supports that plasma fibrinogen concentration was likely increased, given that a negative, moderate and significant relation has been reported between plasma fibrinogen concentration and T_1/2_ among a mixed cohort of participants [27]. Increased magnitude and rate of RBC aggregation is clinically relevant, given that RBC aggregates have an increased effective particle size when compared with single RBC, resulting in significantly increased resistance between adjacent flow streamlines within blood, and also increased resistance to flow [10]. The increased resistance to blood flow has been suggested to be especially detrimental in populations with impaired vascular regulatory mechanisms (e.g., type 2 diabetes; cardiovascular diseases), whereby tissue perfusion may be adversely affected leading to tissue ischaemia.

The primary determinant of high-shear blood viscosity is the degree of RBC deformability, and in the present study, no significant differences in RBC deformability were detected between PCOS and CON (Fig 3). This finding is in close agreement with, and confirms, the present similarity observed between groups for high-shear blood viscosity (Fig 1). The capacity for normal RBC deformability in response to shear stress is altered following exposure to free radicals [10], and given oxidative stress has been widely reported among women with PCOS [12], it was surprising that RBC deformability was not decreased for women with PCOS. It is plausible that the younger age and absence of obesity, and/or a fully functional antioxidant defence system of the present PCOS women, may explain the normal RBC deformability.

The effect of haematocrit on circulatory health has been of clinical interest for some time: the tension between the potentially positive effects of improved oxygen transport “trade-off” against the negative effects of increased blood viscosity [18, 32]. In the present study, we observed significantly decreased HVR (i.e., RBC oxygen transport effectiveness) at all shear rates in women with PCOS (Fig 4). This finding indicates that some compensatory response to blood hyperviscosity may underlie the mildly, but significantly, decreased haematocrit of the women with PCOS in the current study. Crowell and Smith [32] suggested that optimal haematocrit should decrease with vessel diameter to counteract the negative effects of elevated blood viscosity; specifically, it was modelled that optimal haematocrit was lower for hypertension when compared with dilated vessels. It is thus plausible that the decreased HVR in PCOS represents part of a broader vicious cycle including poor oxygen delivery, in addition to decreased vasomotor tone and impaired physical properties of blood cells. Further studies will elucidate the processes connecting blood and vessel health with the heightened risk of metabolic and cardiovascular disorders in PCOS.

The collective findings of the present study indicate that women with PCOS, with a generally unremarkable cardiovascular risk profile based on typical clinical measurements, present with impaired haemorheological parameters that are associated with heightened risk for subsequent cardiovascular events. While the present study indicated that plasma factors (e.g., fibrinogen) may be responsible for some of the observed altered haemorheology, the current study design did not permit direct measurement of this parameter, thus future studies should explore the link between various plasma factors and physical properties of blood. A potential limitation of the present findings may be associated with the use of contraceptives; it is reported that oral contraceptive use mildly increases RBC aggregation in otherwise healthy women [33], although to a much lower extent than that observed in the present findings. Given PCOS is frequently reported to be associated with vascular dysfunction [23–25], impaired haemorheology likely contributes to earlier findings indicating chronic hypoxia/ischaemia in this disorder [34].
